# Inositol Nutritional Supplementation for the Prevention of Gestational Diabetes Mellitus: A Systematic Review and Meta-Analysis of Randomized Controlled Trials

**DOI:** 10.3390/nu14142831

**Published:** 2022-07-09

**Authors:** Jingshu Wei, Jie Yan, Huixia Yang

**Affiliations:** Department of Obstetrics and Gynecology, Peking University First Hospital, Beijing 100034, China; 1610301133@pku.edu.cn (J.W.); yanjie20120303@126.com (J.Y.)

**Keywords:** inositol, myo-inositol, D-chiro-inositol, gestational diabetes mellitus, insulin resistance, randomized controlled trial, meta-analysis

## Abstract

This study was aimed at assessing the efficacy and safety of inositol nutritional supplementation during pregnancy for the prevention of GDM. PubMed, Embase, MEDLINE, and Cochrane library were systematically searched for randomized controlled trails (RCTs) in this field until May 2022. Primary outcomes were the incidence for GDM and plasma glucose levels by oral glucose tolerance test (OGTT). Pooled results were expressed as relative risk (RR) or mean difference (MD) with a 95% confidence interval (95% CI). Seven RCTs with 1321 participants were included in this study. Compared with the control group, 4 g myo-inositol (MI) supplementation per day significantly decreased the incidence of GDM (RR = 0.30, 95% CI (0.18, 0.49), *p* < 0.00001). It significantly decreased the plasma glucose levels of OGTT regarding fasting-glucose OGTT (MD = −4.20, 95% CI (−5.87, −2.54), *p* < 0.00001), 1-h OGTT (MD = −8.75, 95% CI (−12.42, −5.08), *p* < 0.00001), and 2-h OGTT (MD = −8.59, 95% CI (−11.81, −5.83), *p* < 0.00001). It also decreased the need of insulin treatment, and reduced the incidence of preterm delivery and neonatal hypoglycemia. However, no difference was observed between 1.1 g MI per day plus 27.6 mg D-chiro-inositol (DCI) per day and the control group regarding all evaluated results. In conclusion, 4 g MI nutritional supplementation per day during early pregnancy may reduce GDM incidence and severity, therefore may be a practical and safe approach for the prevention of GDM.

## 1. Introduction

Gestational diabetes mellitus (GDM) is one of the most common complications during pregnancy. It is defined as “diabetes diagnosed in the second or third trimester of pregnancy that was not clearly overt diabetes prior to gestation” according to the latest guideline of the American Diabetes Association (ADA) [1]. The International Diabetes Federation (IDF) has reported [2] that the global prevalence of GDM among pregnant women aged 20–49 years was 16.7% in 2021. According to the recommendations of the International Federation of Gynecology and Obstetrics and guidelines of various countries, a 75 g 2-h oral glucose tolerance test is suggested to be performed on all pregnant women between 24- and 28-weeks’ gestation, in order to diagnose and effectively manage GDM [3]. Current evidence indicates that GDM is associated with not only higher risk of pregnancy complications, but also the long-term health of mothers and infants, such as increased risk of developing type 2 diabetes, cardiovascular disease, chronic kidney disease, and cancer for mothers and obesity, overweight, insulin resistance, and delayed neurocognitive development for their offspring [4,5]. Therefore, it is crucial to find safe, effective, and acceptable measures for the prevention of GDM, and nutritional supplementation has been considered as a possible intervention [6].

Inositol, or cyclohexane−1,2,3,4,5,6-hexol, is a polyol which naturally presents in animal and plant cells. Foods that are rich in inositol include cereals, legumes, oil, seeds, and nuts. Inositol exists under nine stereoisomeric forms, of which myo-inositol (MI) and D-chiro-inositol (DCI) are the most common forms in eukaryotic cells. Inositol serves as the structural basis for a variety of second messengers in eukaryotic cells, thus involved in the transduction of several endocrine signals, including follicle stimulating hormone (FSH), thyroid stimulating hormone (TSH), and insulin. By increasing glucose transporter type 4 (GLUT4) translocation to the cell membrane, MI and DCI act as insulin sensitizers, thereby decreasing insulin resistance and plasma glucose levels [7,8].

It has been reported that inositol may reduce hyperinsulinemia and restore ovarian function in women with polycystic ovarian syndrome (PCOS) [9]. As insulin resistance increasing during pregnancy is now recognized as the main pathophysiological mechanism of GDM, it is reasonable to hypothesize that inositol dietary supplementation may have a preventive effect on the development of GDM and its complications.

Over the past ten years, several randomized controlled trails (RCTs) have investigated the effectiveness of inositol supplementation for the prevention of GDM. A Cochran review in 2015 concluded the body of evidence of this topic was of low quality [10]. Since then, other new RCTs have provided more evidence on this topic. Therefore, this systematic review and meta-analysis is performed to assess and summarize the efficacy and safety of inositol nutritional supplementation during pregnancy for the prevention of GDM.

## 2. Materials and Methods

### 2.1. Study Design

This systematic review is organized in accordance with current recommendations of Preferred Reporting Items for Systematic Reviews and Meta-analyses (PRISMA) guidelines [11].

### 2.2. Search Strategy

A literature search was performed in the following databases: PubMed, Embase, MEDLINE, and Cochrane library from inception to May 2022. The search terms used were inositol, myo-inositol, D-chiro-inositol, gestational diabetes mellitus, gestational diabetes, OGTT, plasma glucose level, insulin resistance, randomized controlled trial, and clinical trial. Additional searches were performed among the reference lists of relevant review articles.

### 2.3. Study Selection

The study selection underwent a two-stage process. Firstly, the titles and abstracts were screened. The full tests of all relevant trials were then examined, based on the following inclusion and exclusion criteria. Duplicated studies were removed.

Inclusion criteria:

1. RCTs evaluating the effects of inositol (MI and/or DCI) for preventing GDM during pregnancy.

Exclusion criteria:

1. Pregnant women who were already diagnosed with type 1 diabetes (T1DM), type 2 diabetes mellitus (T2DM), or GDM.
2. Interventions included supplementation other than inositol and folic acid.
3. Did not assess incidence of GDM as outcome.
4. Pilots, protocols, observational studies, reviews, case reports, trials, comments, letters, news, notes, editorial, or conference abstracts.
5. Unable to access the full text of article.

### 2.4. Data Extraction

Data were extracted from the selected RCTs, including first author, year of publication, research site, number of participants, baseline characteristics of participants, detailed intervention protocols, outcome measures, and any reported adverse events. Outcomes that appeared at least in two included studies were extracted for further meta-analysis.

### 2.5. Outcome Measures

Primary outcomes were the incidence for GDM and plasma glucose levels (fasting glucose, 1-h glucose, and 2-h glucose) of the oral glucose tolerance test (OGTT). Secondary outcomes included maternal health outcomes, delivery outcomes, and neonatal health outcomes. Addtionally, side effects associated with the intervention were also evaluated.

Maternal health outcomes measures:

--Need of insulin treatment (%).

--Hypertensive disorders (%).

Delivery outcomes measures:

--Preterm delivery (%): the birth of a baby at earlier than 37 weeks gestational age.

--Cesarean section (%).

--Shoulder dystocia (%).

Neonatal health outcomes measures:

--Birth weight (g).

--Birth weight (%).

--Macrosomia (%): birth weight ≥ 4000 g.

--Neonatal hypoglycemia (%): plasma glucose level of less than 45 mg/dl.

--Neonatal Intensive Care Unit admission (NICU admission) (%).

### 2.6. Risk of Bias and Quality Assessment

The risk of bias was assessed within studies in seven domains, following the criteria outlined in the Cochrane Handbook for Systematic Reviews of Interventions [12]. Discrepancies were resolved by group discussion.

The quality of evidence was evaluated by using the Grading of Recommendations Assessment, Development, and Evaluation (GRADE) approach as outlined in the GRADE handbook [13].

### 2.7. Data Analysis

The meta-analysis estimated the risk ratio (RR) with the 95% confidence interval (CI) for dichotomous variables, and mean difference (MD) with the 95% CI for continuous variables. Heterogeneity was assessed using I^2^ statistics, with an I^2^ between 50 and 90% indicating substantial heterogeneity [11]. All statistical analyses were performed using Review Manager version 5.4.1 (The Cochrane Collaboration, Oxford, UK).

## 3. Results

### 3.1. Literature Search

**Figure 1:**
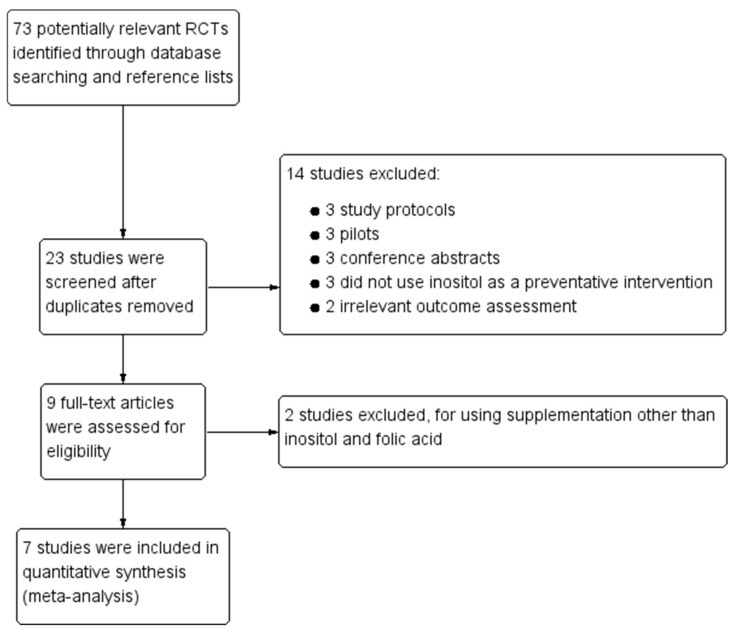
Flowchart of study search and selection.

The initial search in electronic databases and reference lists of review articles identified 23 potentially relevant RCTs. The full text of nine RCTs was retrieved after screaming titles and abstracts, of which seven RCTs were finally included in the meta-analysis, with a total of 1321 patients. The detailed process of the literature search and selection is shown via a flowchart in Figure 1.

### 3.2. Characteristics of Included Studies

The main characteristics of the included studies are summarized in Table 1.

The seven included studies were published between 2013 and 2021, six of which were conducted in Italy, the other one in Ireland. The sample sizes ranged from 73 to 240. Two studies were double-center studies [16,17] and the remaining five were carried out in a single center [14,15,18,19,20].

Two studies [17,19] focused on overweight non-obese women, whose pre-pregnancy body mass index (BMI) was between 25 and 30 kg/m^2^, while one study [16] focused on obese women, whose pre-pregnancy BMI was over 30 kg/m^2^. Two studies [15,18] only included patients with a family history of T2DM. Two studies [14,19] focused on pregnant women with elevated plasma glucose levels in the first trimester.

Six studies [14,15,16,17,19,20] compared the effect of 4 g MI per day with the placebo; two studies [18,19] compared the effect of 1.1 g MI plus 27.6 mg DCI with the placebo; and one study [19] investigated the effect of 500 mg DCI versus the placebo. The placebo was 200 μg folic acid twice a day (or 400 μg per day) in all studies. The duration of intervention in all studies was from trial entry to delivery.

In all studies, the diagnosis of GDM was based on the Hyperglycemia and Adverse Pregnancy Outcome (HAPO) study [21] and the International Association of Diabetes and Pregnancy Study Groups (IADPSG) recommendations [22].

### 3.3. Risk of Bias of Included Studies

**Figure 2:**
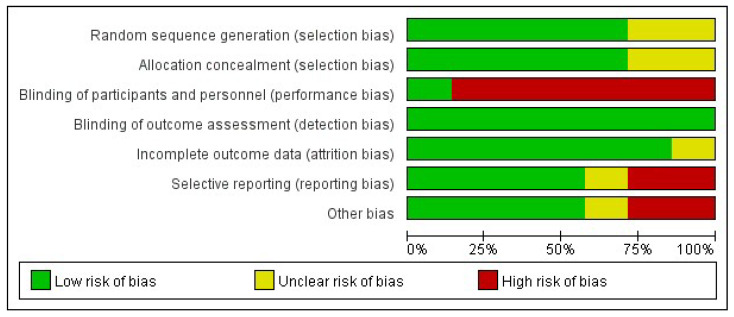
Review of authors’ judgement about each risk of bias item presented as percentages across all included studies.

**Figure 3:**
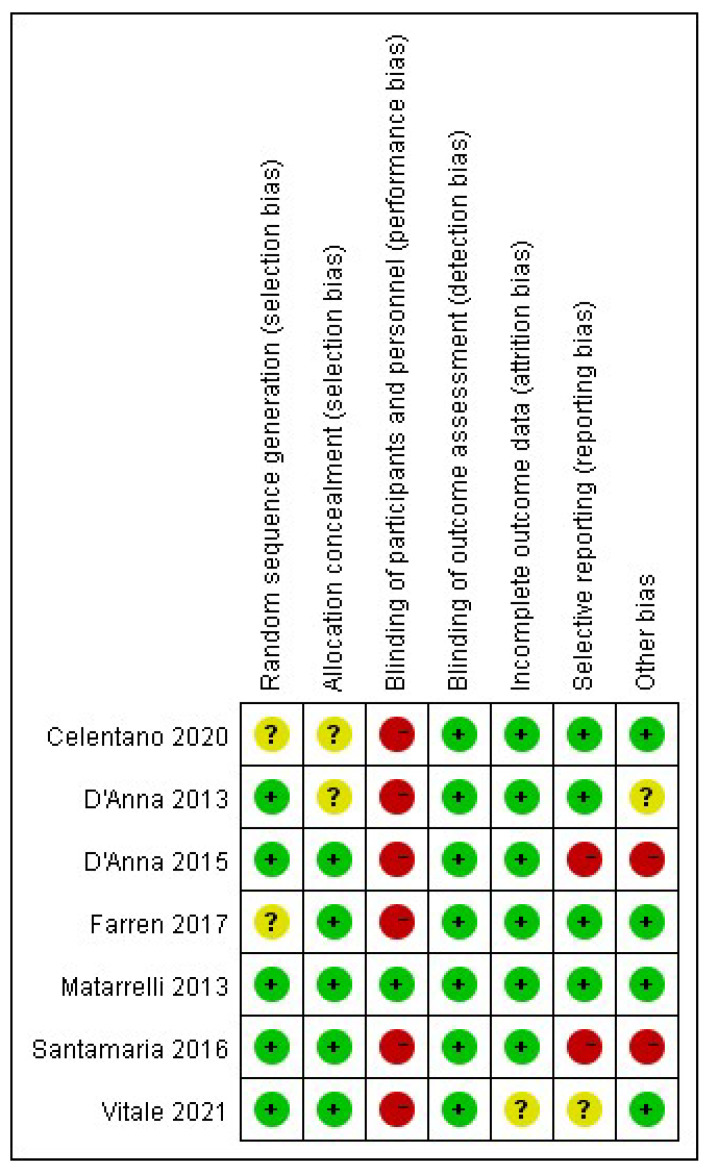
Review of authors’ judgement about each risk of bias item for each included study [14,15,16,17,18,19,20].

The risk of bias of the included studies is shown in Figure 2 and Figure 3.

#### 3.3.1. Selection Bias

All but two studies used a computer-generated random sequence. Farren 2017 and Celentano 2020 stated the participants were randomized but did not provide further information on the method (unclear risk of bias).

D’Anna 2013 and Celentano 2020 did not report the method of allocation concealment (unclear risk of bias), while the remaining studies used an adequate allocation strategy (four with sealed envelopes and one with central allocation).

#### 3.3.2. Performance Bias

Matarrelli 2013 is the only study which was double-blinded for both participants and healthcare providers. The others were open-label studies and therefore were assessed as having a high risk of bias.

#### 3.3.3. Detection Bias

The outcomes evaluated in these studies were either objective measurements of laboratory values or established facts, which were unlikely to be influenced by the blinding strategy. Therefore, all studies were assessed as having a low risk of bias.

#### 3.3.4. Attrition Bias

None of the seven studies had a drop-out rate over 20%. However, Vitale 2021 used a protocol treatment analysis, therefore was assessed as having an unclear risk of bias.

#### 3.3.5. Reporting Bias

D’Anna 2015 and Santamaria 2016 were assessed as having a high risk of bias because the outcomes were changed after protocol registration. Vitale 2021 was assessed as having an unclear risk of bias because the detailed results of several secondary outcomes were not reported. The remaining studies were assessed as having a low risk of bias.

#### 3.3.6. Other Bias

D’Anna 2015 and Santamaria 2016 were assessed as having a high risk of bias because a baseline difference in the rate of family history of T2DM between groups was reported, which might affect the effect evaluation. D’Anna 2013 stated that an intention-to-treat analysis was performed and did not show results different from those of per-protocol analysis, but only published the results of per-protocol analysis. Therefore, it was assessed as having an unclear risk of bias. The remaining studies were assessed as having a low risk of bias.

### 3.4. Effects of Intervention

#### 3.4.1. Primary Outcomes

All seven studies evaluated the incidence of GDM and OGTT plasma glucose levels.

**Figure 4:**
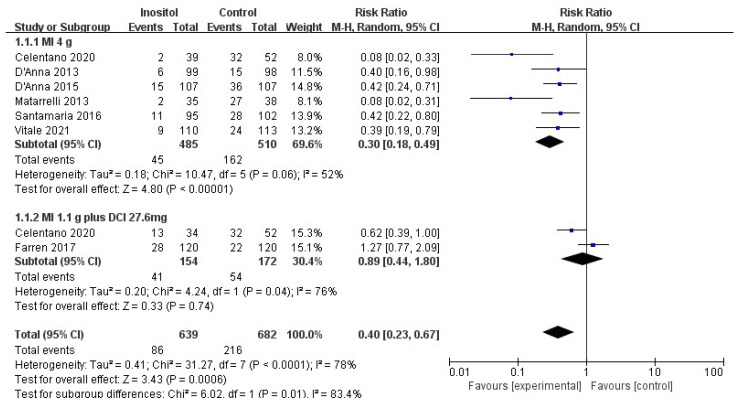
Inositol vs. control: incidence of GDM [14,15,16,17,18,19,20].

Compared with the control group, 4 g MI supplementation per day significantly decreased the incidence of GDM (RR = 0.30, 95% CI (0.18, 0.49), *p* < 0.00001; six trials, *n* = 995), with substantial heterogeneity among studies (I^2^ = 52%, *p* = 0.06). No difference was observed between 1.1 g MI plus 27.6 mg DCI supplementation per day and the control group (*p* = 0.74; two trials, *n* = 326) (Figure 4).

**Figure 5:**
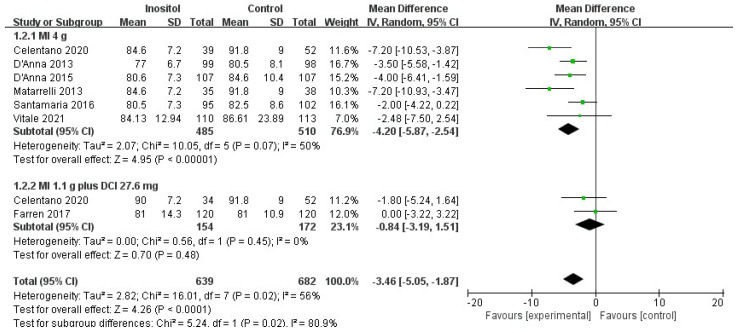
Inositol vs. control: fasting-glucose OGTT [14,15,16,17,18,19,20].

**Figure 6:**
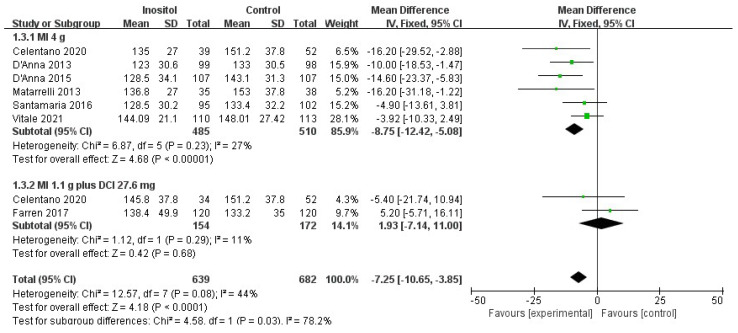
Inositol vs. control: 1-h OGTT [14,15,16,17,18,19,20].

**Figure 7:**
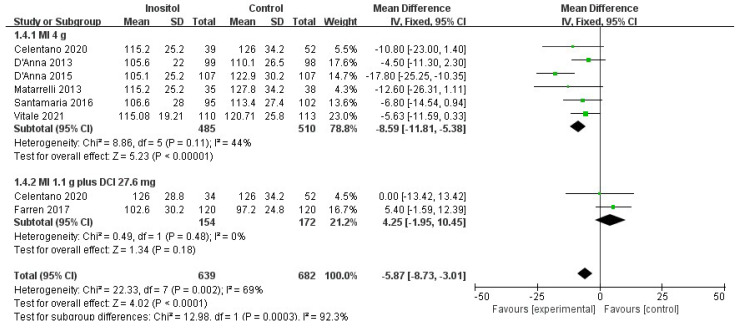
Inositol vs. control: 2-h OGTT [14,15,16,17,18,19,20].

Compared with the control group, 4 g MI supplementation per day significantly decreased the OGTT plasma glucose levels regarding fasting-glucose OGTT (MD = −4.20, 95% CI (−5.87, −2.54), *p* < 0.00001, I^2^ = 50%; six trials, *n* = 995) (Figure 5), 1-h OGTT (MD = −8.75, 95% CI [−12.42, −5.08], *p* < 0.00001, I^2^ = 27%; six trials, *n* = 995) (Figure 6), and 2-h OGTT (MD = −8.59, 95% CI (−11.81, −5.83), *p* < 0.00001, I^2^ = 44%; six trials, *n* = 995) (Figure 7). No difference was observed between 1.1 g MI plus 27.6 mg DCI supplementation per day and the control group (*p* = 0.45/0.29/0.48; two trials, *n* = 326).

#### 3.4.2. Secondary Outcomes

Four studies evaluated patients’ need of insulin treatment. Compared with the control group, 4 g MI supplementation per day significantly decreased the need of insulin treatment (RR = 0.27, 95% CI (0.11, 0.66), *p* = 0.004, I^2^ = 0%; four trials, *n* = 562).

Five studies evaluated the incidence of hypertensive disorders. No difference in the incidence of hypertensive disorders was observed between 4 g MI supplementation per day and the control group or 1.1 g MI plus 27.6 mg DCI supplementation per day and the control group (respectively, *p* = 0.06, four trials, *n* = 686; *p* = 0.23, two trials, *n* = 320).

Five studies evaluated the incidence of preterm delivery. Compared with the control group, 4 g MI supplementation per day significantly decreased the incidence of preterm delivery (RR = 0.39, 95% CI (0.18, 0.82), *p* = 0.01, I^2^ = 0%; four trials, *n* = 686). No difference was observed between 1.1 g MI plus 27.6 mg DCI supplementation per day and the control group (*p* = 0.13; two trials, *n* = 320).

Five studies evaluated the cesarean section rate. No difference in the cesarean section rate was observed between 4 g MI supplementation per day and the control group or 1.1 g MI plus 27.6 mg DCI supplementation per day and the control group (respectively, *p* = 0.14, four trials, *n* = 686; *p* = 0.57, two trials, *n* = 320).

Three studies evaluated the incidence of shoulder dystocia. No difference in the incidence of shoulder dystocia was observed between 4 g MI supplementation per day and the control group (*p* = 0.48, three trials, *n* = 595).

Six studies evaluated neonatal birth weight. No difference in birth weight was observed between 4 g MI supplementation and the control group or 1.1 g MI plus 27.6 mg DCI supplementation per day and the control group (respectively, *p* = 0.12, five trials, *n* = 759; *p* = 0.88, two trials, *n* = 320). However, compared with the control group, 4 g MI supplementation per day significantly decreased birth weight in percentiles (MD = −14.86, 95% CI (−21.38, −8.35), *p* < 0.00001, I^2^ = 0%; two trials, *n* = 164).

Three studies evaluated the incidence of macrosomia. No difference in the incidence of macrosomia was observed between 4 g MI supplementation per day and the control group (*p* = 0.22, three trials, *n* = 595).

Six studies evaluated the incidence of neonatal hypoglycemia. Compared with the control group, 4 g MI supplementation per day significantly decreased the incidence of neonatal hypoglycemia (RR = 0.15, 95% CI (0.03, 0.66), *p* = 0.01, I^2^ = 0%; five trials, *n* = 759). No difference was observed between 1.1 g MI plus 27.6 mg DCI supplementation per day and the control group (*p* = 0.53; two trials, *n* = 320).

Four studies evaluated the incidence of NICU admission. No difference in the NICU admission rate was observed between 4 g MI supplementation per day and the control group or 1.1 g MI plus 27.6 mg DCI supplementation per day and the control group (respectively, *p* = 0.08, three trials, *n* = 489; *p* = 0.75, two trials, *n* = 320).

### 3.5. Side Effects

A total of 1321 patients from seven studies were included in the meta-analysis, among which no side effect was reported.

### 3.6. Overall Quality of Evidence

The overall quality of evidence was rated as very low or very low for all the outcomes evaluated (Table 2).

## 4. Discussion

The incidence of GDM has been increasing worldwide over the past years. As current evidence indicates that GDM is associated with higher risk of pregnancy complications and long-term maternal and fetal adverse outcomes, researchers have been focused on finding more practical measures to prevent GDM. Clinical trials and observational studies have shown that exercise can be a non-invasive therapeutic option [23,24], while others have focused on diet control and nutritional supplementation.

Inositol is a polyol which widely exists in eukaryotic cells. It is the structural basis of various secondary messengers, especially of inositol triphosphates, phosphatidylinositol phosphate lipids, and inositol glycans. Therefore, inositol is involved in insulin signal transduction through regulating the phosphatidylinositol 3-kinase (PI3K)/protein kinase B (AKT) signal pathway and increasing GLUT4 translocation to the cell membrane, which has been supported by the latest animal experiments in type 2 diabetic db/db mice [25]. It is natural to consider inositol as an effective insulin sensitizer to prevent GDM, the main pathophysiological mechanism of which is believed to be insulin resistance.

The present meta-analysis included seven RCTs with a total number of 1321 participants. In all, 485 patients received the intervention of 4 g MI supplementation per day, and 154 patients received the intervention of 1.1 g MI plus 27.6 mg DCI supplementation per day. The results suggest that compared with the control group (placebo), 4 g MI supplementation per day during pregnancy significantly reduced incidence of GDM, plasma glucose levels of OGTT, and the need of insulin treatment. It was also associated with significantly reduced incidence of preterm delivery, lower birth weight (in percentiles), and reduced incidence of neonatal hypoglycemia. The incidence of hypertensive disorders and NICU admission rate also reduced, although not statistically significant. It showed no remarkable impact on the incidence of cesarean section, shoulder dystocia, and macrosomia. It is noted that no difference was observed between 1.1 g MI plus 27.6 mg DCI supplementation per day and the control group in all outcomes. The dosing regimen for MI+DCI was extrapolated from previous studies investigating the effects of inositol in women with PCOS [9]. However, this dosage seemed inadequate in terms of preventing GDM.

The present meta-analysis included RCTs that used inositol as the only intervention. There were also studies investigating the effect of inositol plus other nutritional supplementation. Dell’Edera 2017 [26] suggested that a combination of 1.75 g MI, 250 mg DCI, plus zinc and methylsulfonylmethane might prevent the onset of GDM in pregnant women with glucose intolerance. Godfrey 2021 [27], however, found the combination of 4 g MI plus vitamin D/B6/B12, riboflavin, zinc, and probiotics was not effective in an international, multi-center, double-blind trial. Previous studies have suggested that vitamin D supplementation could possibly reduce the risk of GDM [28], while the effect of probiotics remained unclear [29]. Whether the other components of the intervention played an additional role is yet to be investigated. Melvasi 2014 [30] and Melvasi 2017 [31] focused on the improvement of maternal metabolic profile, suggesting that the intervention of 2 g MI plus 400 mg DCI, and several other components could improve glycemic and lipidic parameters in pregnant women. In addition, 400 μg folic acid supplementation per day was given to all participants as a placebo control in the RCTs included in this meta-analysis. Several cohort studies have reported that folic acid supplementation in early pregnancy [32] and prolonged duration [33] of it may increase the risk of GDM. This is unlikely to affect the results of the present meta-analysis but should be taken into consideration when conducting further clinical trials in the future.

Inositol is included in the list of compounds that are ‘generally recognized as safe’, according to United States Food and Drug Administration, which means it has been proven to be free of side effects and is safe for use in pregnancy [34]. Among the studies included in the present meta-analysis, no adverse event associated with inositol supplementation was reported, and previous reviews declared the same conclusion [35]. Therefore, as a safe molecule at usual administered dosage, inositol could be a potential choice of nutritional supplementation for preventing GDM.

Previous systematic reviews and meta-analyses mostly focused on the effects of MI and suggested that MI supplementation during pregnancy shows the potential in preventing GDM, with low quality previous evidence [10,36,37,38]. The main conclusion is similar between the present study and previous studies. However, the presented meta-analysis has included all existing RCTs with the intervention of inositol only (both MI and DCI) on the topic up to May 2022. Three aspects of a secondary outcome were evaluated, including ten subitems. A more comprehensive evaluation of inositol’s effect on GDM prevention was performed by the present study.

Still, several limitations should be taken into consideration. Six studies were conducted in Italy with Caucasian women. The lack of a blinding method in most studies lead to an overall high risk of performance bias, and the risk of reporting bias was unclear. Moreover, there was substantial heterogeneity when performing a sensitivity analysis of some outcomes. Therefore, the overall quality of evidence was rated as low or very low for all the outcomes evaluated. Further studies are required in different countries and ethnic groups. The dosage of inositol and intervention time need to be further evaluated. Besides, long-term outcomes of both mothers and their offspring should be included in the evaluation.

## 5. Conclusions

Inositol nutritional supplementation of 4 g MI per day during early pregnancy may reduce GDM incidence and severity. It may also decrease the need of insulin treatment, and reduce the incidence of preterm delivery and neonatal hypoglycemia. Besides, it is reported to be safe for use in pregnancy. Therefore, inositol nutritional supplementation may be a practical and safe approach for the prevention of GDM. Further studies are required to apply inositol into more clinical practice.

## Figures and Tables

**Table 1:** The main characteristics of the included studies. BMI: body mass index. GA: gestational age. FPG: fast plasma glucose. RG: random glucose.

Study	Country	Inclusion Criteria	Interventions	Inositol Group	Control Group
Matarrelli 2013 [14]	Italy	(1) Prepregnancy BMI < 35 kg/m^2^ (2) FPG ≥ 5.1 mmol/L and <7.0 mmol/L in the first or early second trimester (3) Single pregnancy	Group A: 2 g MI plus 200 μg folic acid twice a day Group B: 200 μg folic acid twice a day	Group A (*n* = 35) Age: 33.0 ± 4.9 BMI: 23.5 ± 3.4 FPG: 5.4 ± 0.3	Group B (*n* = 38) Age: 33.8 ± 4.7 BMI: 24.7 ± 4.2 FPG: 5.4 ± 0.3
D’Anna 2013 [15]	Italy	(1) GA: 12~13 w (2) First-degree relatives affected by T2DM (3) Prepregnancy BMI < 30 kg/m^2^ (4) FPG < 126 mg/dl, RG < 200 mg/dl (5) Single pregnancy (6) Caucasian race	Group A: 2 g MI plus 200 μg folic acid twice a day Group B: 200 μg folic acid twice a day	Group A (*n* = 99) Age: 31.0 ± 5.3 BMI: 22.8 ± 3.1	Group B (*n* = 98) Age: 31.6 ± 5.6 BMI: 23.6 ± 3.1
D’Anna 2015 [16]	Italy	(1) GA: 12~13 w (2) Prepregnancy BMI ≥ 30 kg/m^2^ (3) FPG < 126 mg/dl, RG < 200 mg/dl (4) Single pregnancy (5) Caucasian race	Group A: 2 g MI plus 200 μg folic acid twice a day Group B: 200 μg folic acid twice a day	Group A (*n* = 107) Age: 30.9 (18~44) BMI: 33.8 (30.0~46.9) FPG: 83.1 ± 8.5	Group B (*n* = 107) Age: 31.7 (19~43) BMI: 33.8 (30.0~46.0) FPG: 82.3 ± 10.6
Santamaria 2016 [17]	Italy	(1) GA: 12~13 w (2) Prepregnancy BMI ≥ 25 kg/m^2^, <30 kg/m^2^ (3) FPG < 126 mg/dl, RG < 200 mg/dl (4) Single pregnancy (5) Caucasian race	Group A: 2 g MI plus 200 μg folic acid twice a day Group B: 200 μg folic acid twice a day	Group A (*n* = 95) Age: 32.1 ± 4.8 BMI: 26.9 ± 1.3 FPG: 81.09 ± 8..03	Group B (*n* = 102) Age: 32.7 ± 5.3 BMI: 27.1 ± 1.3 FPG: 78.63 ± 6.15
Farren 2017 [18]	Ireland	(1) GA: 10~16 w (2) First-degree relatives affected by T1DM/T2DM (3) FPG < 126 mg/dl, RG < 200 mg/dl (4) Single pregnancy	Group A: 1.1 g MI, 27.6 mg DCI plus 400 μg folic acid per day Group B: 400 μg folic acid per day	Group A (*n* = 120) Age: 31.1 ± 5.1 BMI: 26.0 ± 5.3	Group B (*n* = 120) Age: 31.5 ± 5.0 BMI: 26.2 ± 5.5
Celentano 2020 [19]	Italy	(1) Prepregnancy BMI < 35 kg/m^2^ (2) FPG ≥ 5.1 mmol/L and <7.0 mmol/L in the first trimester (3) Single pregnancy	Group A: 2 g MI plus 200 μg folic acid twice a day Group B: 500 mg DCI plus 400 μg folic acid per day Group C: 0.55 g MI, 13.8 mg DCI plus 200 μg folic acid twice a day Group D: 400 μg folic acid per day	Group A (*n* = 39) Age: 33.1 ± 4.9 BMI: 23.5 ± 3.4 FPG: 5.4 ± 0.3 Group B (*n* = 32) Age: 34.4 ± 3.7 BMI: 24.4 ± 4.9 FPG: 5.3 ± 0.2 Group C (*n* = 34) Age: 34.1 ± 4.2 BMI: 23.5 ± 4.6 FPG: 5.4 ± 0.3	Group D (*n* = 52) Age: 33.9 ± 4.9 BMI: 24.4 ± 4.1 FPG: 5.4 ± 0.3
Vitale 2021 [20]	Italy	(1) GA: 12~13 w (2) Prepregnancy BMI ≥ 25 kg/m^2^, <30 kg/m^2^ (3) FPG < 126 mg/dl, RG < 200 mg/dl (4) Single pregnancy (5) Caucasian race	Group A: 2 g MI plus 200 μg folic acid twice a day Group B: 200 μg folic acid twice a day	Group A (*n* = 110) Age: 27.18 ± 6.03 BMI: 27.00 ± 1.49 FPG: 82.20 ± 12.12	Group B (*n* = 113) Age: 27.95 ± 4.90 BMI: 26.68 ± 1.56 FPG: 83.10 ± 14.10

**Table 2:** Summary of finding: inositol for the prevention of GDM.

Inositol for the Prevention of GDM						
Patient or Population: Women in Early Pregnancy Who Were at Risk of GDM (Those with Pre-Existing T1DM/T2DM Excluded) Intervention: MI 4 g/MI 1.1 g Plus DCI 27.6 mg						
Outcomes	Illustrative comparative risks * (95% CI)		Relative effect (95% CI)	No of Participants (studies)	Quality of the evidence (GRADE)	Comments
	Assumed risk	Corresponding risk				
	Control	Inositol				
GDM rate	Study population		RR 0.4 (0.23 to 0.67)	1321 (7 studies)	⊕⊖⊖⊖ very low ^1,2,3,4^	
	317 per 1000	127 per 1000 (73 to 212)				
	Moderate					
	306 per 1000	122 per 1000 (70 to 205)				
Insulin treatment	Study population		RR 0.27 (0.11 to 0.66)	562 (4 studies)	⊕⊕⊖⊖ low^ 1,2,4,5^	
	84 per 1000	23 per 1000 (9 to 56)				
	Moderate					
	106 per 1000	29 per 1000 (12 to 70)				
Hypertensive disorders	Study population		RR 0.43 (0.2 to 0.91)	1006 (5 studies)	⊕⊖⊖⊖ very low ^1,2,4^	
	42 per 1000	18 per 1000 (8 to 38)				
	Moderate					
	30 per 1000	13 per 1000 (6 to 27)				
Preterm delivery	Study population		RR 0.4 (0.22 to 0.74)	1006 (5 studies)	⊕⊖⊖⊖ very low ^1,2,4^	
	69 per 1000	27 per 1000 (15 to 51)				
	Moderate					
	63 per 1000	25 per 1000 (14 to 47)				
Cesarean section	Study population		RR 0.89 (0.77 to 1.03)	1006 (5 studies)	⊕⊖⊖⊖ very low ^1,2,4,6^	
	448 per 1000	398 per 1000 (345 to 461)				
	Moderate					
	471 per 1000	419 per 1000 (363 to 485)				
Shoulder dystocia	Study population		RR 0.58 (0.12 to 2.68)	595 (3 studies)	⊕⊖⊖⊖ very low ^1,2,4,6^	
	13 per 1000	8 per 1000 (2 to 35)				
	Moderate					
	10 per 1000	6 per 1000 (1 to 27)				
Macrosomia	Study population		RR 0.35 (0.06 to 1.92)	595 (3 studies)	⊕⊖⊖⊖ very low ^1,2,3,4,6^	
	56 per 1000	20 per 1000 (3 to 107)				
	Moderate					
	49 per 1000	17 per 1000 (3 to 94)				
Neonatal hypoglycemia	Study population		RR 0.62 (0.32 to 1.18)	1079 (6 studies)	⊕⊖⊖⊖ very low ^1,4,6^	
	46 per 1000	29 per 1000 (15 to 54)				
	Moderate					
	10 per 1000	6 per 1000 (3 to 12)				
NICU admission	Study population		RR 0.53 (0.23 to 1.21)	809 (4 studies)	⊕⊖⊖⊖ very low ^1,2,4,6^	
	37 per 1000	20 per 1000 (9 to 45)				
	Moderate					
	39 per 1000	21 per 1000 (9 to 47)				

⊕ Upgrade. ⊖ Downgrade. ^1^ Downgraded due to the high risk of performance bias and unclear risk of reporting bias. ^2^ Downgraded because the research location and race of participants were limited. ^3^ Downgraded due to high heterogeneity. ^4^ Publication bias due to “positive results” is strongly suspected. ^5^ Upgraded because the RR/*p* value is low. ^6^ Downgraded due to wide CI crossing the line of no effect. * The basis for the assumed risk (e.g., the median control group risk across studies) is provided in footnotes. The corresponding risk (and its 95% confidence interval) is based on the assumed risk in the comparison group and the relative effect of the intervention (and its 95% CI).
